# Cybersecurity Analysis of Wearable Devices: Smartwatches Passive Attack

**DOI:** 10.3390/s23125438

**Published:** 2023-06-08

**Authors:** Alejandra Guadalupe Silva-Trujillo, Mauricio Jacobo González González, Luis Pablo Rocha Pérez, Luis Javier García Villalba

**Affiliations:** 1 Facultad de Ingeniería, Universidad Autónoma de San Luis Potosí (UASLP), Zona Universitaria, San Luis Potosí 78290, Mexico; 2 Facultad de Informática, Group of Analysis, Security and Systems (GASS), Department of Software Engineering and Artificial Intelligence (DISIA), Faculty of Computer Science and Engineering, Office 431, Universidad Complutense de Madrid (UCM), 28040 Madrid, Spain; 3 Instituto Tecnológico de Estudios Superiores de Monterrey, Escuela de Ingeniería y Ciencias, Departamento de Computación, Campus Puebla, Puebla 72453, Mexico

**Keywords:** Bluetooth, BLE, cybersecurity, secure connections, smartwatch, sniffer, wearable

## Abstract

Wearable devices are starting to gain popularity, which means that a large portion of the population is starting to acquire these products. This kind of technology comes with a lot of advantages, as it simplifies different tasks people do daily. However, as they recollect sensitive data, they are starting to be targets for cybercriminals. The number of attacks on wearable devices forces manufacturers to improve the security of these devices to protect them. Many vulnerabilities have appeared in communication protocols, specifically Bluetooth. We focus on understanding the Bluetooth protocol and what countermeasures have been applied during their updated versions to solve the most common security problems. We have performed a passive attack on six different smartwatches to discover their vulnerabilities during the pairing process. Furthermore, we have developed a proposal of requirements needed for maximum security of wearable devices, as well as the minimum requirements needed to have a secure pairing process between two devices via Bluetooth.

## 1. Introduction

Internet of Things technologies are evolving and taking part in our daily routines without us even noticing [1]. The continuous growth and acceptance of these devices are going out of proportion, as the new normal shows a person owning multiple IoT devices. It is projected that by the year 2025, there will be over 75 billion connected devices [2]. Furthermore, just five years later, by 2030, it is expected that there will be 124 billion IoT devices [3].

**Figure 1:**
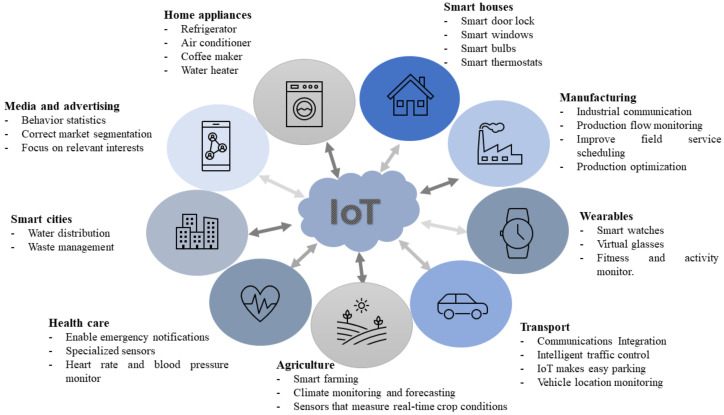
IoT Application Areas.

IoT reaches different scopes; they can be medicine, education, industry, entertainment, sports, clothes, smart cities, agriculture, and many others. See Figure 1. Technology recollects a big amount of data, including personal information, routines, and health records, to simplify the diverse tasks that we accomplish daily. However, having that great collection of records could be counterproductive, if someone else uses it to gain something. This opens the door for cybercriminals, who understand the value of these types of sensitive data.

IoT device cyberattacks are deemed to be of high risk, particularly when handling people’s health data, as they can lead to physical harm and endanger lives. Vulnerabilities not only impact device functionality, but also people’s health [4]. These devices are anticipated to have high demand in the market, and manufacturers strive to optimize their components for cost reduction and focus on providing minimum functionality, disregarding fundamental security needs. Furthermore, a considerable number of device manufacturers do not provide software updates or security patches to mitigate or prevent damage after an attack.

Researchers have reported that over 68,000 medical devices were identified in Shodan to be exposed and therefore accessed on the public internet. Some of the devices were Magnetic Resonance Imaging (MRI) machines, infusion pumps, and pacemaker systems. These devices had default configuration settings. Researchers were able to extract some information related to office numbers, employee names, default credentials, software versions, operating systems, and more [5]. In some cases, the attackers did not realize what devices they were infecting. If they had acknowledged it, they would have been able to get a lot of sensitive information and could have caused damage to the hospital’s IT infrastructure.

Doctors are now able to program implantable cardioverter-defibrillators (ICD) to monitor a patient’s heart condition. These devices can send the right level of electrical shock to get the heart beating properly [6]. It was found out how attackers could cause a malfunction in these devices provoking a dangerous shock in the patient.

The COVID-19 pandemic has made IoT technologies increasingly valuable as they provide numerous benefits such as communication, work, staying updated on daily events, learning, entertainment, health monitoring, and promoting a healthy lifestyle [7]. By reducing medical costs and encouraging healthy habits, IoT devices have become more popular due to the growing demand from people worldwide. Given the importance of monitoring individuals’ health to prevent potential consequences, IoT technologies can play a crucial role in detection, especially in situations where continuous monitoring is necessary to prevent putting a larger group of people at risk [8,9].

Data privacy will be a very important point that will have to be established because if it gets compromised, it will cause individuals to reject this type of technology. The increase in devices also makes these gadgets a target for an attacker. Thus, it will be a great challenge to have the necessary protection for each person’s data. Different strategies started to appear involving COVID-19 and smart devices. Effective contact tracing adds importance to the user’s privacy. Trying to identify individuals who have been exposed to an infected person during the contagious window while preserving our privacy [10]. Wearable devices include a large list of possible attacks [11]. While there are multiple ways to mitigate these threats, the continuous advance of these technologies also introduces some challenges to countermeasure these vulnerabilities [12,13]. Likewise, wearable devices, similar to smartwatches, come with a great number of limitations that must be addressed [14,15] to increase public acceptance of this kind of technology. As these types of devices are gaining popularity, for this project, we have focused on a cybersecurity analysis of smartwatches due to the diverse amount of data they obtain as they are worn all day. These devices recollect data such as location, messages, phone calls, and medical information such as heart rate. Some types of smartwatches also collect temperature and oxygen saturation (SpO2); the multiple uses they have, as they can be used to track exercise activities and sleeping activity; and the acceptance they have received in the latter years by the public, as we see every day more smartwatch consumers. Furthermore, even when a smartwatch is not formally considered a medical device, today’s wearable devices are more than just human activity trackers. Several types of research have shown that smartwatch users are taking their activity notifications seriously as a tool for health management. Even more, there has been a phenomenon of users feeling stress or anxiety about health readings from smartwatches and other digital wellness tools [16]. A study of young people showed that they track and monitor their bodies and health behaviors as a common practice [17,18,19].

Concerns have arisen regarding the lack of comprehension related to data security and privacy, as well as the vulnerability of these trackers to various attacks. With the increasing prevalence of personal health data disclosure and breaches in wearable devices, it is essential to further investigate the security and privacy challenges associated with these devices to provide a better, more secure, and private user experience. Users should be aware that these devices may present data inaccuracies and should be considered only as a tool rather than something entirely accurate for making health decisions.

This paper focuses on the vulnerabilities of smartwatches during their pairing via Bluetooth with other devices. Bluetooth has been a victim of different attacks for many years. In Section 2, we present several studies in which the authors describe vulnerabilities and various types of attacks on different smartwatches, as well as providing proposals to manufacturers and users for mitigating these threats. Section 3 describes the Bluetooth protocol, and how it has been evolving. We have also included the security recommendations proposed by the Bluetooth guide [20]. Section 3 also exhibits the difference between pairing methods on Bluetooth devices, exposing the weakest and the safest methods. Section 4 describes how the cybersecurity analysis has been developed as well as our findings on the smartwatches that have been tested. Section 5 presents a proposal for maximum-security requirements and a proposal for the minimum requirements needed for security, which have been made with the most necessary security features while pairing two devices via Bluetooth. Section 6 expresses the conclusions gathered during this project and our future work.

## 2. IoT Vulnerabilities and Challenges

The literature shows multiple concerns already found in different IoT devices [21]. Now, to understand how big these concerns are, vulnerabilities must be covered due to the increasing use of this kind of technology and the sensitive information they gather. A key is to maintain confidentiality, integrity, and availability, also known as the CIA triad; this represents a fundamental concept in cybersecurity. There has been a lot of research, and the most common types of attacks that have been made on IoT devices can be seen in the systematic review in Table 1.

The large number of attacks that exist on IoT devices gives us an idea of the importance of establishing security countermeasures against these threats [24]. The consequences that they can have on a person’s lifestyle and health could be devastating. Devices’ sensitive data could be at risk with a technique in which data is being sent through devices and because they have poor authentication methods for devices that handle such an important type of data, raising question marks about confidentiality.

Other types of attacks have the objective of changing data information, making the users’ data that was recollected hard to trust. This way, it damages the integrity part of the IoT device. Several IoT devices are now used for medical purposes, recollecting real-time information. If they are not available at every moment of the day, not only are they not fulfilling their purpose, but they could be putting a user’s life in danger by not registering, what might be, for some users, life and death cases.

As can be seen, the biggest fundamentals in cybersecurity have been exposed by these types of technologies. Correcting these problems would be the following step to take to guarantee confidentiality, integrity, and availability. However, numerous other issues appear when trying to apply new forms of security to IoT devices, as shown in Table 2.

### Smartwatches: Bluetooth Communication Attacks

In this paper, we are focused on the Design Constraints. One of the main technologies used in the IoT for device communication is Bluetooth. Bluetooth is everywhere: in speakers, headphones, refrigerators, cars, wearables, medical devices, and more. IoT is about small devices and multiple sensors. Bluetooth is a suitable technology that provides IoT features that can be applied to a wide range of potential IoT applications. Manufacturers need to understand how to implement secure architectures to protect users’ sensitive information while considering the challenges and limitations of the IoT.

However, Bluetooth communication has been the target of multiple types of attacks for years as cybercriminals exploit the vulnerabilities this technology has had in earlier versions. These communication protocols have been updated to protect devices against eavesdropping and man-in-the-middle attacks.

The literature shows multiple researchers finding weaknesses in wearable devices [29,30,31,32]. They talk about different attacks on several IoT devices that communicate via Bluetooth, as well as they give countermeasures and recommendations to users for safer use of this technology. Furthermore, other research includes studies that found vulnerabilities in some devices [33]. One of them is an attack on a smartwatch, where the PIN that secures its communication with a smartphone was exploited while performing a brute-force attack. In the pairing process, this smartwatch had one of the least secure mechanisms, which shows that smartwatches are prone to attacks [34]. With respect to threats, one article divides them into two categories: passive adversary and active adversary, where the first, the attacker eavedrops on the connection and tries to find LTK or other information, but he can not manipulate the message, and in the second, the attacker can inject, modify, and block the message transmitted, enabling the ability to create his own messages and send them to the victim’s device [35].

Other research focuses on wearable devices, such as a Fitbit smartwatch, and how they can be targets of man-in-the-middle attacks. The attack consists of using two fake devices, one that disguises itself as a smart device and another one as a mobile app that connects to the Fitbit device; it also adds that Fitbit collects a lot of sensitive data, and it proposes to educate the users to be aware of what happens when doing an incorrect use of the device [36]. Another research project highlights a couple of vulnerabilities in some wearable devices, such as the smartwatch Fitbit Inspire, which has a serious threat known as the Link Layer Length Overflow. It means that an attacker acting as a central device can make the peripheral devices suffer instabilities until they finally crash [37].

More studies have concluded that it is important to teach users about the correct use of this technology because most of the recommendations always go to the manufacturers. They propose some guidelines to instruct about wearable devices [38] and state the need for standards in the wearable industry [36].

Another piece of research worries about the data these devices obtain; for example, the users’ location, which exposes them to different types of attacks. Furthermore, it proposes the constant change of the MAC address to avoid any type of targeting [39]. A group of researchers also mentions the vulnerabilities of the MAC address in Fitbit devices, as they recollect the MAC addresses of nearby Fitbit devices, and while Fitbit offers a reasonable level of security, they also gather extraneous data about users [40].

In one paper, they make passive attacks on wearable devices using Bluetooth sniffers and HCI snoop logs and capture an encryption key in plain text [41]. While another article shows the use of Bluetooth and describes an attack where it forces a key renegotiation using eavesdropping techniques [42]. Other works show the potential risks the devices are exposed to when manufacturers do not follow the recommendations of the Bluetooth Special Interest Group, as it happens more often than it should [43,44]. While various research projects include tests on different devices, the literature does not show a way to compare the security levels of these devices. To the best of our knowledge, there is no existence of measurement to identify when an IoT device is secure or what characteristics can be considered to categorize if an IoT device is safe or not.

At this point, we can summarize our contribution as follows:

- We proposed a novel model of Security Requirements on smartwatches in order to categorize if a device is safe or not based on the Bluetooth guide [20].
- We performed an analysis of six smartwatches to evaluate their security requirements and showed the comparison.
- We establish a proposal for maximum and minimum security designs for smartwatches.

## 3. Background: Bluetooth Technologies and Evolution

Bluetooth is used for short-range radio-frequency communication. As mentioned before, vulnerabilities can be found in IoT devices, and this could be discovered through the Bluetooth protocol. The most common attacks are man-in-the-middle (MITM), where an attacker can obtain the keys that are exchanged between devices and, once obtained, eavesdrop on communications [45].

**Figure 2:**
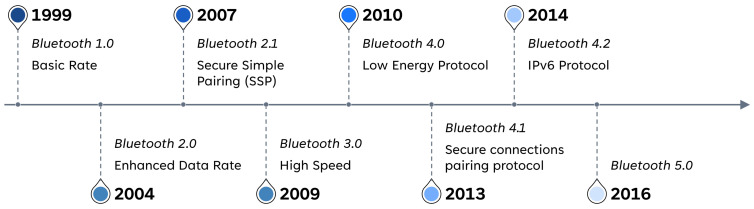
Bluetooth Technologies and Evolution.

The earliest days of Bluetooth introduced Bluetooth Basic Rate (BR), Enhanced Data Rate (EDR), and High Speed (HS) models. Bluetooth 1.1 and 1.2 versions could only work with BR because they are only capable of supporting up to 1 megabit per second (Mbps). EDR improves in Bluetooth version 2.0, where it gets data rates up to 3.0 Mbps. HS arrives during Bluetooth 3.0, supporting faster data rates up to 24 Mbps. However, devices that support higher data rates are also able to support lower data rates from earlier Bluetooth specifications. When referring to these versions of Bluetooth, they are commonly known as Bluetooth Classic. See Figure 2.

Bluetooth Low-Energy (BLE) was established in the Bluetooth 4.0 specification; later, an update was made in versions 4.1 and 4.2. It is useful for wearable medical devices and sensors because it was primarily made for devices that use a coin-cell battery. It reduces power consumption and memory requirements. Basically, it is designed to operate in sleep mode and wake up only when the connection is initiated. This improves efficiency when discovering devices and during connection procedures and results in packets with shorter lengths, while services and protocols are simpler.

Since Bluetooth 4.0 devices can support both Bluetooth Classic and BLE, this is known as the dual mode. Cellphones work as a perfect example, where they might use Bluetooth Classic when connected to earphones and have the necessity to have constant data streaming while also using BLE when connected to a smart wristband that tracks your activity while doing exercises, and you only need the data exchange when you synchronize your devices to check your results.

Bluetooth has five basic security services:

(a) Authentication, using the Bluetooth address to verify the identity of each device during the communication stage.
(b) Confidentiality, guaranteeing that only authorized devices have access to data, avoiding any type of eavesdropping.
(c) Authorization, verifying that a device is authorized to use the service before allowing it to do it, guaranteeing that only this device can use the service and no other device can.
(d) Message integrity, when information is exchanged between two Bluetooth devices, it has to be secure and nothing can be modified.
(e) Pairing/bonding, the generated keys are shared and stored for future use, to create trust between two Bluetooth devices.

To understand the importance of the keys that are exchanged once two devices start pairing, we have to understand the Bluetooth protocols and the security levels to avoid eavesdropping during this process. We are going to discuss these security levels and modes for each Bluetooth specification, first Bluetooth Classic and later Bluetooth Low-Energy.

### 3.1. Bluetooth Classic

Bluetooth includes four security modes; mode 1 has no security, mode 2 has authentication and encryption in the controller, and mode 3 has it in the physical link. These three modes only existed in the prior Bluetooth 2.1 version. In this article, we only test communication between devices that have a Bluetooth version higher than the Bluetooth 2.1 version. For these devices, it is mandatory to work with security mode 4.

Security Mode 4 is a service-level enforced security mode; it uses secure simple pairing (SSP) and it uses Elliptic-curve Diffie–Hellman (ECDH) key agreement for link key generation. This helps protect against eavesdropping and man-in-the-middle attacks. The ECDH that is used could be the elliptic curve 192 or 256. For authentication and encryption, a secret symmetric key is necessary, and it is known as the link key. Security mode 4 includes five security levels. Starting from security level 0 and ending at security level 4: (i) Level 0 has no security and is only allowed for service discovery protocols; (ii) Level 1, also does not require security; (iii) Level 2 requires an unauthenticated link key; (iv) Level 3 requires an authenticated link key, and; (v) Level 4 requires authenticating the link key using secure connections. The secure connections pairing protocol was introduced in Bluetooth 4.1 and it uses the ECDH 256, improving from the ECDH 192 that was used prior.

### 3.2. Bluetooth Low Energy

This section meticulously explains BLE, to understand how it is possible to protect against the most common attacks on this technology.

Bluetooth 4.0, 4.1, and 4.2 count cryptographic keys to improve security in the devices, these keys are named Identity Resolving Key (IRK), to support low-energy private device addresses, and Connections Signature Resolving Key (CSRK), to assist data signing. When pairing BLE devices, a Long-Term Key (LTK) is generated, which is important for authentication and encryption (known as the link key in Bluetooth Classic). This could result in two different methods. During the first method, one device generates the LTK and sends it to the other device in a secure manner; this is known as low-energy Legacy Pairing. Furthermore, it is important to notice that for this method, all the keys are distributed in a secure process during the pairing stage. In the second method, both devices create the key without the need to share it through the link. This method is called low-energy Secure Connections. Meanwhile, the LTK is generated, while the IRK and the CSRK are created and distributed securely. An important difference between these methods is that low-energy Legacy Pairing does not include Elliptic-curve Diffie–Hellman (ECDH) encryption, which makes it vulnerable to eavesdropping attacks and allows attackers to potentially find the LTK. In contrast, Low-energy Secure Connections can countermeasure this threat. We will provide a more detailed review of these pairing methods later in this paper.

Low-energy Security includes two modes. Security Mode 1 has four levels related to encryption. Level 1 does not require encryption and authentication. Level 2 asks for unauthenticated pairing with encryption. Level 3 needs authenticated pairing with encryption. Level 4 uses the Secure Connections method previously discussed in this section, as it asks for an authenticated link key using low-energy Secure Connections pairing with encryption. Security Mode 2 requires data signing in both of its levels, with the sole difference that level 1 only needs unauthenticated pairing while level 2 asks for authenticated pairing. Because encryption is a great security asset, using Security Mode 1 Level 3 or 4 is strongly recommended over other options.

### 3.3. Bluetooth: Pairing Methods

In this section, we give a more detailed explanation of the low-energy pairing methods and describe the phases that occurred during the pairing methods. Starting with low-energy Legacy Pairing. Phases:

(i) Phase 1, once explore the input/output capabilities and security requirements in the devices, they will establish an agreement on a Temporary Key (TK).
(ii) Phase 2, they proceed to create a Short Term Key (STK) using random values that are being exchanged and the TK. This STK establishes an encrypted link between devices.
(iii) Phase 3, it assures a safe key transport for all the keys mentioned earlier in this article (LTK, IRK, CSRK).

Low-energy Secure Connections work in a different manner. Even though phase 1 works the same way as legacy pairing, in phase 2, the LTK is generated without the need for the STK. This LTK is useful in phase 3, and the LTK encrypts the links, and a key agreement is made to distribute the IRK and CSRK securely instead of using a key transport.

During the pairing process between two devices, one of four different pairing processes can be applied. These pairing processes are: (a) Out of Band for Bluetooth Standard or BLE; (b) Numeric Comparison; (c) Passkey Entry 4 or 6 digits; and (d) Just Works. The input/output capabilities of devices play an important role in determining what processes can be utilized.

The out-of-band (OOB) process requires two devices that have OOB technology, such as near-field communication (NFC). One device sends the other a 128-bit number, which is the TK, using OOB technology. Using low-energy Legacy Pairing provides one-in-a-million protection against MITM attacks to guess the TK. However, this protection comes from the OOB technology that the device uses, because if someone is capable of eavesdropping on the OOB, they will obtain the TK values. For low-energy Secure Connections, the device address is sent through the OOB. Even if an eavesdropper can obtain it, this does not give them any value in decrypting the data.

Numeric comparison is an option available only for low-energy Secure Connections. This method is not available for low-energy Legacy Pairing. The process involves two devices displaying a 6-digit number on their respective screens, and the user enters one of two options: (i) YES, if both displays show the same 6-digit number; (ii) NO, if the numbers are different. The previous 6-digit number is not used to generate the link key to avoid eavesdropping. Even if an unauthorized person captures this 6-digit number, it will not be useful for any further pairing process. Additionally, it has protection against MITM attacks, as the user must confirm if the 6-digit number is or is not the same on both devices before proceeding. This guarantees that no other device can initiate the pairing process.

Another method is passkey entry, which requires that both devices have a keyboard input or at least one of them has a display output. This method works with low-energy Legacy Pairing. A passkey is given on one device and entered on the other, and then a TK is generated using the passkey. The passkey is required to have six numeric digits, which would give an entropy of twenty bits that ensures the complexity of deciphering the given key. Low-energy Secure Connections pairing works differently. After the devices exchange public keys, the six numeric digits passkey is generated, and once it is entered into the device, it starts sending a hash of each bit of the passkey. This procedure is repeated twenty times to complete the twenty bits of the passkey. Furthermore, the public keys were sent during the previous step. This method offers protection against MITM attacks. When using a passkey of six digits, it gives an attacker a one-in-a-million chance to guess the correct passkey.

The last method is the least secure one, and it is used due to the limitations in the input/output capabilities of the devices. For low-energy Legacy Pairing the key is always the same and is set to all zeros, leaving the pairing exposed to eavesdropping and MITM attacks. In the low-energy Secure Connections method, the pairing process follows the same steps as in the numeric comparison process, but the user is unable to see the 6-digit number. This is because the devices involved in this procedure cannot display the number, which in turn makes it impossible to perform the final commitment checks.

**Figure 3:**
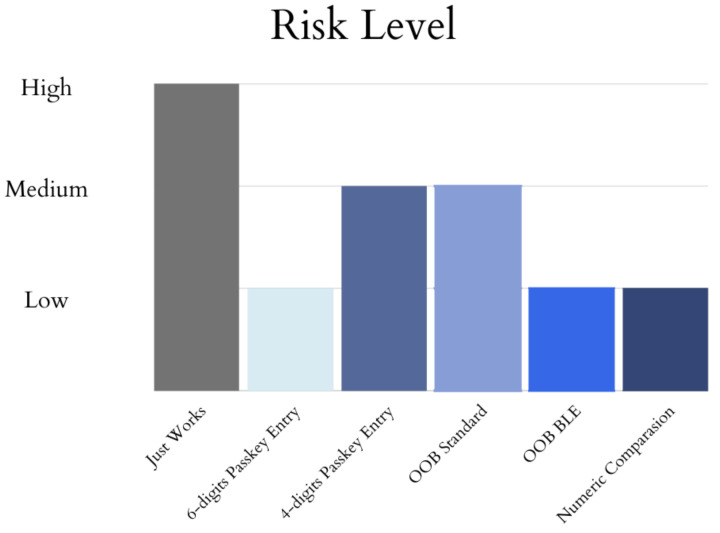
Risk Level on Pairing Methods.

These four pairing methods are not exclusive to Bluetooth Low-Energy; they can also be found in Bluetooth Classic, working slightly differently due to the IRK and CSRK being exclusive to BLE. Only the LTK is set to be created, but it is known as the link key. The association models (out of band, numeric comparison, and passkey entry) provide authenticated link keys; meanwhile, the link key is unauthenticated during the Just Works pairing model for Bluetooth Classic. The risk of an attack is determined by the version of Bluetooth and the pairing method used. See Figure 3.

## 4. Passive Attack: An Traffic Analysis on Smartwatches

### 4.1. Threat Model

In this paper, we consider a passive attacker who does not interfere with the pairing process and only eavesdrops on the exchanged communication packets. This adversary is also called an honest-but-curious or passive global attacker because he or she can see all the traffic communication in the system.

### 4.2. Data Source

For this project, we aimed to exploit the vulnerabilities of the Bluetooth protocol in wearable devices due to the increase in use that they are showing in the general public. We chose to study various types of smartwatches from different brands. The limitations they might have in hardware and their input/output capabilities gave us reasons to believe that they could be unable to follow every step of the Bluetooth protocol guidelines [20].

**Figure 4:**
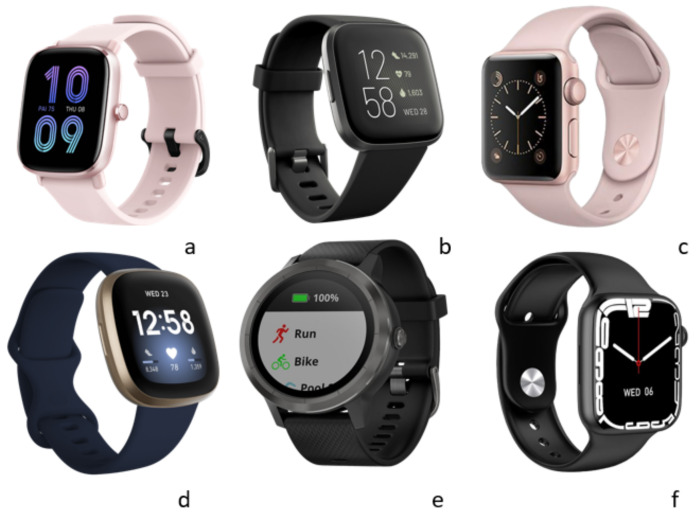
Smartwatches used in the attack; (**a**) Amazfit GTS 2 mini; (**b**) Fitbit Versa 2; (**c**) Apple Watch Series 2 Aluminum; (**d**) Fitbit Versa 3; (**e**) Garmin vivoactive 3; (**f**) W27 Pro.

For the experiment, we used six smartwatches. We carefully selected six devices that we believe would provide a good representation of the current market. We acknowledge that there are many other devices available, but we believe that our selection provides a useful and informative sample for our research purposes. See Figure 4:

Smartwatches are under consideration for medical diagnosis. For example, in 2018, the Food and Drug Administration (FDA) cleared the Apple Watch Series 4 and named it a class 2 medical device [46] because of its ability to identify atrial fibrillation (AF). This shows that manufacturers are designing smartwatches that can obtain sensitive data, and because no standard has to be followed to design these devices, they create a world of possibilities for cybercriminals. We tested six different smartwatches; all of them include the heart rate detection function, while others also include blood oxygen saturation (SpO2) detection. These features could encourage the user to seek medical advice when necessary and could save multiple lives; for these reasons, it is important to guarantee the confidentiality, integrity, and availability of these data for smartwatch users.

### 4.3. Analysis and Findings

**Figure 5:**
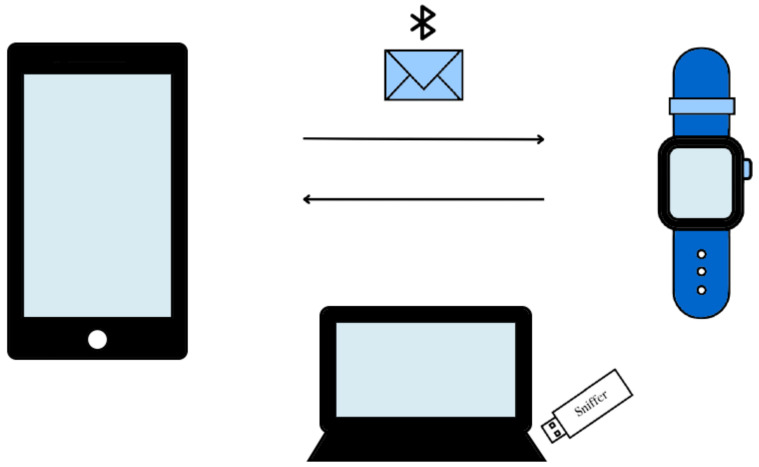
Passive Attack: Threat model.

To test the security features during the Bluetooth pairing process of these devices, we implemented a passive sniffing attack where we captured the traffic sent between devices. We used two Bluefruit LE Sniffer - Bluetooth Low-Energy (BLE 4.0)-nRF51822-Firmware Version 2, designed by adafruit. It allowed us to listen to only BLE devices and capture their traffic. It is recommended to use at least two sniffers, ideally three, because the packets are sent through 40 frequency channels (3 for advertising and 37 for data), and normally one packet is sent over different channels, and only one sniffer could capture them all, causing loss of information. Once we obtained it, we started analyzing the data packets using wireshark, an open-source packet analyzer. We made our attacks in a Dell-Inspiron laptop 14 serie 7000, intel core i7 with Kali Linux. See Figure 5.

First, all the packets were captured while pairing a smartwatch and a smartphone, we noticed all packets sent over the Attribute Protocol (ATT) were missing; normally all the data exchange is here, whether encrypted or unencrypted. We found that once the smartwatches bonded with a smartphone, the sniffer stopped capturing data from the devices because their connection was encrypted thanks to the key exchange or agreement they had during pairing. We explored other ways to analyze the pairing process between our devices. Except for the Apple Watch Series 2, all the devices were paired with a Samsung Galaxy S20. This smartphone has Bluetooth 5.0 dual mode, which allows it to connect to devices with BLE and Bluetooth Classic. It also has the feature to generate a Bluetooth host controller interface (HCI) snoop log, which gives us the option to obtain records of the Bluetooth data that our smartphone is generating while pairing with other devices.

There are two ways to obtain the HCI log, and it depends on the smartphone and its operating system. For the one we selected, we had to generate a bug report. To do this, we activated the developer mode and extracted all the activity and bugs from the smartphone during a previous period of time. The report was stored in a .txt file extension on our device. However, this file contained information that was not useful for our purpose, so we used the btsnooz python script to extract only the captured Bluetooth information.

(1) btnooz.pyoriginalbugreport.txt>newbluetoothsnoop.log

The other way to obtain the bug report is also by enabling developer mode, activating the Bluetooth HCI snoop log option, and finally rebooting the smartphone. This is supposed to automatically generate the report more easily. Nevertheless, in our case, this file was never generated. We tried other devices, such as a Xiaomi Mi9T smartphone and a Huawei MatePad tablet, but had no success with either of them. We believe that this method is no longer available.

With our analysis, we were able to find that the way different smartwatches work during pairing and the lack of standardization for wearable design show multiple differences inside the security scope. Table 3 includes the devices that were analyzed during this project and their features. We show the Bluetooth version of each device and whether their pairing method is in the Bluetooth Classic mode or the BLE mode.

**Figure 6:**
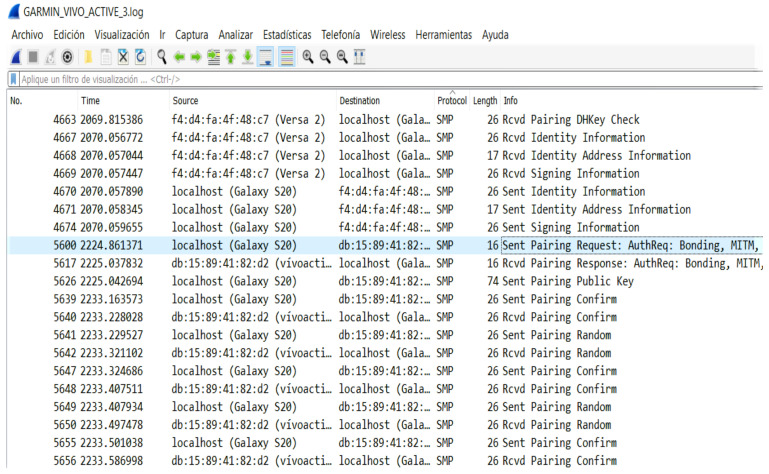
Process of pairing authentication of Garmin vivoactive 3.

For the Fitbit Versa 3 smartwatch, we discovered that it uses Bluetooth dual mode, as it has a feature that enables users to make and receive calls called Versa 3 Controls. To use this feature, the smartwatch must first be paired with the user’s smartphone via BLE. Once this is done, another pairing must be initiated via Bluetooth Classic between the smartphone and Versa 3 Controls to utilize the phone’s features for making and receiving calls. For this device, we decided to separate this feature and list it as another device due to the requirement of performing another pairing process to use it. Although it is one physical device, we are now counting it as two different smartwatches, one pairing via BLE and the other via Bluetooth Classic. Therefore, we have analyzed an extra device, resulting in a total of seven smartwatches being tested. For the Garmin, we found that besides showing the 6-digit number on both devices during the pairing process, when we analyzed the data packets in Wireshark, we noticed that the pairing authentication was performed repeatedly, much more than the other smartwatches, as shown in Figure 6. This is because it uses the LE Secure Connections method, in which each device computes a confirmation value and sends it to the other device; then this second device also computes its own confirmation value and sends it to the first device, iterating for each k bit of the passkey. Table 3 also shows the pairing association model these devices use; sometimes they include more than one model. The reason is to be able to pair to smartphones with different input/output capabilities. These parameters are shown in the data traffic within the Security Manager Protocol when both devices share I/O capabilities when sending the pairing request. Some of these association models are less secure than others.

We can see the security modes and their respective levels previously discussed in this paper.

Another feature that we noticed during the testing of these devices is that some of the smartwatches have a static address, which could be quickly identified, revealing the type of device and its version. To test this vulnerability, we used an open-source framework called HomePwn, which allowed us to use this information and exploit the vulnerability. Unlike using a sniffer to capture packets, this method only requires the Bluetooth capability of a laptop. This framework has many modules that we can load and use, but we only considered the Bluetooth low-energy and classic Bluetooth modules. These show active Bluetooth devices and some information, such as the Received Signal Strength Indicator (RSSI) and MAC address. Some devices also show the name of the manufacturer and the device name. This easily allows cybercriminals to discover the objective and gather more information about the device.

We can summarize our findings as:

1. Six of seven smartwatches pair using a Bluetooth protocol 4.0 or higher.
2. Five of them utilize low-energy pairing.
3. Two of them do not utilize an authentication pairing.
4. Six of them have a static address.

In general, all smartwatches present vulnerabilities that can be exploited by attackers to carry out different types of attacks. The first opportunity for attack is through passive sniffing, which involves intercepting packets during communication between two devices. Attackers can collect this information to exploit the weak pairing protocols of the devices. Passive sniffing can be successful when attacking devices with low-security pairing protocols, such as those using the Just Works association model. Our tests were conducted during the pairing process, but attackers can use other techniques to achieve successful passive sniffing even when two devices are already paired, such as device cloning, jamming, and injection-free techniques [47]. These methods work by forcing two devices to unpair, which results in a renegotiation of their keys; this allows the attacker to sniff the communication channels to obtain information. Furthermore, these methods have some limitations, as it is necessary for the attacker to stay close to the target while performing the attacks [48]. Other attacks that are possible after carrying out a successful passive sniffing are man-in-the-middle attacks, offline pin cracking attacks, or fuzzing attacks; one of these methods would work depending on the weakness found during the passive sniffing attack.

## 5. Proposed Model to Achieve Security and Privacy on Smartwatches

**Figure 7:**
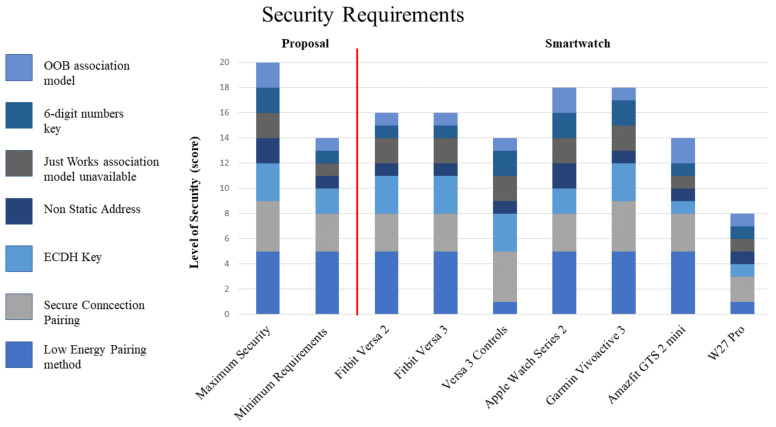
Security requirements comparison for smartwatches. The maximum security can go up to twenty points, and the minimum score to have a secure device is equal to fourteen.

After understanding how these smartwatches work during pairing, we started to propose a model for the maximum-security requirements identified that a wearable device must be included while connecting via Bluetooth. Furthermore, taking into account the many countermeasures that the Bluetooth standard has applied to the most common attacks such as eavesdropping and man-in-the-middle. It is important to note that there is a minimum set of security requirements that must be included in every wearable device to be able to take care of these attacks. For this reason, Figure 7 includes a proposal to meet these requirements to accomplish the minimum standard of security. We get these results from a metric that we created, and we assign points to every security feature that is covered. However, every feature has a different number of points, as we consider some features to be more important than others. As it is noticed in Figure 7, the maximum security has a total of twenty points, and for the minimum requirements, the total is 14 points; this would mean the device is considered secure according to our metrics. The features of our proposal are the following:

(a) Low-Energy Pairing method. This feature is at the base of our scale because it is the most important feature we consider for this research. Smartwatches are wearable devices that must use the BLE protocol to achieve their ideal functionality, which makes this feature essential. We assigned five points to the smartwatches that include this feature and only one point to the devices that do not.
(b) Secure Connections Pairing. As mentioned earlier, secure connections are the most secure pairing procedure, and they were introduced in Bluetooth version 4.1 for Bluetooth Classic and in version 4.2 for BLE. This has significant weight in our scale; nevertheless, lower security methods such as authenticated pairing and encryption, while they do not offer protection against eavesdropping, are better protocols and are recommended for their use instead of unauthenticated pairing, which also does not offer man-in-the-middle protection. We assign the points according to the security level that can be found in Table 3. Level 4 gets four points because it is a higher security level, while level 1 gets one point for being a lower security level.
(c) ECDH Key. ECDH-based cryptography also offers protection against eavesdroppers. Our proposal for minimum security and our proposal for maximum security show a slight difference due to the existence of two methods for ECDH-based cryptography. In order to gain the maximum security grade, it must work with a P-256 elliptic curve, while other methods might use a P-192 elliptic curve that still offers protection against eavesdrop attacks. For the case of the P-256 elliptic curve, three points are assigned; the P-192 elliptic curve gets two points; and devices without this feature only get one point in our metric.
(d) Non-Static Address, even if there is no change in the address, it could attract the interest of different attackers. We do not have a big impact on this feature for our scale because, in the BLE pairing process, the IRK helps to countermeasure attacks that aim to exploit the address of the device. However, changing the address occasionally would give our devices the best protection against other types of attacks. For these reasons, we only assign two points to devices with this feature and one point to those without it.
(e) The Just Works association model is unavailable; when referring to the association models, it is important to notice that Just Works is the least secure. This is commonly used when a device or both devices do not have the input/output capabilities required to pair using another method or when the information exchange is not sensitive, such as with headphones or speakers. For the best security practices, smartwatches should not include this feature and instead use another association model. However, if they include this pairing process, the user should be responsible and choose the safest pairing association model. For this case, we assign two points to devices that do not include this pairing method, and we assign one point to devices that include it, because it could be just another method in the device, and the user can choose not to pair the device using this method.
(f) 6-digit numbers key. This feature is present in two association models: passkey entry and numeric comparison. It is important to note that the use of a 6-digit passkey is required for maximum security when using secure connections method. However, some devices only include a 4-digit passkey. While this difference may seem significant when using the passkey entry model, it is worth mentioning that this number is not used to generate any security key. Therefore, even if a cybercriminal were to obtain this number, it would not be useful for carrying out any eavesdropping attacks on the smartwatch. We assign two points in our metric only when the key is a 6-digit number; if it is shorter, or the device does not include this method, we assign one point.
(g) OOB association model. For BLE legacy pairing, this association model is considered the most secure. Although it is not necessary for the minimum security requirements proposal, as we just mentioned, for devices that work with BLE legacy pairing, such as the Apple Watch Series 2, it offers the maximum security that a device could have during pairing. For this reason, we assign two points when this pairing association model is included. However, we only penalize it with one point when it is not included.

## 6. Conclusions and Future Work

### 6.1. Conclusions

The increase in popularity of wearable devices and the continuous adoption by a large portion of the population of this technology to track their daily routines require that the manufacturers of these devices can ensure the confidentiality, integrity, and availability of the data that is being gathered. Some of the most recent smartwatches collect sensitive data, such as health information, in an accurate manner that allows users to gain trust in these devices and know when to reach for a medical consultation.

The method we used to attack the devices is passive; it aids to understand which devices would be the best targets to exploit their weak pairing protocols and start to read real-time information about the users. As these technologies keep growing and adding sensors, the attacker would be able to obtain sensitive real-time data and create a profile of a possible victim.

Protection against all types of cyberattacks is vital for upcoming technology. The guide to Bluetooth security gives recommendations to have the maximum security for this communication protocol, but as we showed during our cybersecurity study, those guidelines are not always followed entirely. Some modifications are made to accomplish the main objectives during the design of the devices. The tests exhibited that some devices do not include the most recent security protocol even if they work with the newest Bluetooth version, as seen when comparing the Fitbit Versa 2 and Fitbit Versa 3. The first one uses the Bluetooth 4.0 version; for this reason, it does not work with the safest pairing protocol as it has not been created yet. Meanwhile, the Fitbit Versa 3 includes the Bluetooth 5.0 version and still uses the same security protocol as its previous version. Only one out of six smartwatches tested did not meet all the necessary requirements, as shown in Figure 7. Therefore, buying a new device is not a guarantee that it will come with better security and privacy features. However, it is obvious that the higher the price of the device, the higher the level of security it has, such as with the Garmin Vivoactive 3 and Apple Watch Series 2. Both devices showed the greatest scores on our proposal for security requirements.

Another device showed that it does not pair with the BLE protocol, ignoring the recommendations given. Not only for better security but also for better usability.

Our proposal for minimum requirements necessary to establish a secure pairing between two devices is useful to reveal which smartwatches should not be on the market due to a lack of security. We can notice that 16% of the analyzed devices were not in the approved range by our metric.

This proposal could be a step forward in the creation of new regulations that aim to assure the security of wearable technology. Our analysis should also be taken into account by manufacturers that design smartwatches, as completing the maximum security for devices is not their primary goal. This metric helps accomplish the minimum security requirements needed. Furthermore, it could help users understand when a device is not secure and differentiate when a less secure pairing method is applied.

We also want to point out the importance of having a legal framework for IoT devices that forces vendors to follow security and privacy requirements in order to make a new IoT product. A law that mandates security and privacy rules for IoT devices ensures that all devices adhere to a minimum set of security and privacy standards. It is out of the scope of our paper, but we think it is relevant to establish the reasons for further work in this area, such as (i) Protection Consumer Privacy; (ii) Preventing Cyberattacks; (iii) Promoting industry standards; (iv) Encouraging innovation; (v) Building trust. A customer is more likely to consume and use an IoT device if he knows that his personal data is being handled in a secure and responsible manner. It is known that part of the responsibility for the information also depends on the consumer; smartwatch users must be aware of the risk of sharing personal or sensitive data with another device. In this case, if a smartwatch works with the Just Works association models, it is probably because that data is unencrypted. However, we know that there is a lot of ignorance about how pairing authentication and data exchange work between a smartphone and a smartwatch, and at the end of the day, it is difficult for a non-knowledgeable user to understand this process, so the vendors also need to do their job by ensuring that devices will comply with the confidentiality and integrity of the user’s information.

### 6.2. Future Work

Based on our findings, we know that there are a lot of IoT devices on the market that do not have the basic requirements to follow security and privacy rules. Therefore, we would like to explore and analyze other kinds of IoT medical devices such as electrocardiogram (ECG) Monitoring systems and Brain Computer Interface (BCI) systems. As we mentioned in this paper, smartwatches are not entirely considered medical devices, but we can notice a growing adoption of IoT technology to help reduce operational costs and improve healthier habits for people. With the rapid and unstoppable integration of multiple technologies into the medical field, it is expected that people will adopt more IoT devices. We have not found much research on this topic, evaluating medical devices used on a daily basis by users, and we believe our findings and expertise in this area could be applied to experiments on medical IoT devices, such as EGC and BCI.

## Figures and Tables

**Table 1:** Systematic Review: IoT Attacks.

IoT Attacks	Research			
	[22]	[23]	[24]	[25]
Eavesdropping	✓	✓		✓
Traffic Analysis	✓	✓		✓
Information Gathering	✓			
Modification	✓		✓	✓
Masquerade	✓			
Denial of Service	✓	✓	✓	✓
Replay	✓	✓		✓
Based on Network Properties		✓	✓	✓
Malevolent Code				✓
Phishing				✓

**Table 2:** Systematic Review: Security Challenges.

Challenges	Research				
	[22]	[24]	[26]	[27]	[28]
Computational Limitations	✓	✓			
Memory Limitations		✓			
Energy Limitations	✓	✓	✓		
Mobility		✓	✓		
Scalability		✓		✓	
Communications Media		✓	✓	✓	
Multiplicity of Devices		✓			
Dynamic Network Topology	✓	✓			
Multi-protocol Network		✓			
Dynamic Security Updates		✓	✓		
Tamper-Resistant Packages		✓			
Design Constraints			✓		
Price				✓	✓

**Table 3:** Bluetooth features of the analyzed devices.

Device Name	Bluetooth Protocol	Low Energy Pairing	Pairing Methods	Pairing Association Model	Passkey	Security Mode	Level	Static Address
Fitbit Versa 2	4.0	✓	LE Legacy Pairing	Passkey Entry	4 digits	1	3	✓
Fitbit Versa 3	5.0	✓	LE Legacy Pairing	Passkey Entry	4 digits	1	3	✓
Versa 3 Controls	5.0	×	Secure Simple Pairing	Numeric Comparison	6 digits	4	4	✓
Apple Watch Series 2	4.0	✓	LE Legacy Pairing	Passkey Entry or OOB	6 digits	1	3	×
Garmin Vivoactive 3	4.2	✓	LE Secure Connections	Passkey Entry	6 digits	1	4	✓
Amazfit GTS 2 mini	5.0	✓	LE Legacy Pairing	OOB or Just Works	None	1	3	✓
W27 Pro	3.0 + 5.0	×	Secure Simple Pairing	Just Works	None	4	2	✓

## Data Availability

Not applicable; this study does not report any data.
